# A Manual of Chemistry

**Published:** 1846-08

**Authors:** 


### ARTICLE IX.

*A Manual of Chemistry.* By Richard D. Hoblin, A. M., OXON., author of a "Dictionary of terms used in Medicine and the collateral sciences." New York: Samuel S. and William Wood, 126 Pearl street. 1846. pp. 335. (From the Publishers.)

The manual before us, is, in our opinion, one of the best yet published for the use of students just beginning the study of Chemistry.

It seems to have been the object of the author, to state all the most important facts and theories of the science, in as clear a light and in as condensed a form as possible, and, as he remarks, "to make the student acquainted with the facts which Chemistry is daily presenting to our notice; to enable him from the consideration of these facts, to contemplate the laws which regulate the economy of nature; to stimulate him to pursue the science even into its farthest recesses."

We cordially recommend the book, as well adapted to the wants of students, beginning the study of this important science. Teachers in schools and acadamies, will find it a most valuable text book. W. B. H.

